# No Evidence of a Genetic Causal Relationship between Ankylosing Spondylitis and Gut Microbiota: A Two-Sample Mendelian Randomization Study

**DOI:** 10.3390/nu15041057

**Published:** 2023-02-20

**Authors:** Mingyi Yang, Xianjie Wan, Haishi Zheng, Ke Xu, Jiale Xie, Hui Yu, Jiachen Wang, Peng Xu

**Affiliations:** Department of Joint Surgery, HongHui Hospital, Xi’an Jiaotong University, Xi’an 710054, China

**Keywords:** ankylosing spondylitis, gut microbiota, genetic, causal, mendelian randomization

## Abstract

**Objective:** Ankylosing spondylitis (AS) is associated with a variety of gut microbiotas. We aim to analyze the causal relationship between the two at the genetic level. Methods: Mendelian randomization (MR) is a type of instrumental variables (IVs) analysis; MR follows the Mendelian genetic rule of “parental alleles are randomly assigned to offspring” and takes genetic variation as IVs to infer the causal association between exposure factors and study outcome in observational studies. Genome-wide association study (GWAS) summary data of AS were from the FinnGen consortium, and the gut microbiota (Bacteroides, Streptococcus, Proteobacteria, Lachnospiraceae) were from the MiBioGen consortium. The TwoSampleMR and MRPRESSO packages of the R were used to perform a two-sample MR study. Random-effects inverse variance weighted (IVW) was the main analysis method, and MR Egger, weighted median, simple mode, and weighted mode were used as supplementary methods. We examined heterogeneity and horizontal pleiotropy, and examined whether the analysis results were influenced by a single SNP. We applied radial variants of the IVW and MR-Egger model for the improved visualization of the causal estimate. We further examined the causal relationship between AS and gut microbiota, and the robustness of the analysis results. Finally, we performed maximum likelihood, penalized weighted median, and IVW (fixed effects) to further identify the potential causal association. **Results:** The random-effects IVW results showed that Bacteroides (*p* = 0.965, OR 95% confidence interval [CI] = 0.990 [0.621–1.579]), Streptococcus (*p* = 0.591, OR 95% CI = 1.120 [0.741–1.692]), Proteobacteria (*p* = 0.522, OR 95% CI = 1.160 [0.737–1.826]), and Lachnospiraceae (*p* = 0.717, OR 95% CI = 1.073 [0.732–1.574]) have no genetic causal relationship with AS. There was no heterogeneity, horizontal pleiotropy or outliers, and results were normally distributed. The MR analysis results were not driven by a single SNP. **Conclusions:** This study showed that Bacteroides, Streptococcus, Proteobacteria and Lachnospiraceae, four common gut microbiotas associated with AS, had no causal relationship with AS at the genetic level. This study makes a positive contribution to the genetics of AS, but the insufficient number of gut microbiota included is a limitation.

## 1. Introduction

Ankylosing spondylitis (AS) is a chronic and progressive disease which mainly affects the sacroiliac joint, spine, para-spinal soft tissue and peripheral joints, and may be accompanied by extra-articular manifestations. The main clinical manifestations are waist, back, neck and hip pain and joint swelling pain. In severe cases, spinal deformity and joint ankylosis may occur [1]. AS is widely distributed all over the world, and the incidence varies greatly in different regions and different populations [2]. Studies have found that the incidence of AS may be related to genetics, infection, immunity and other factors. The major histocompatibility complex (MHC) haplotype Human Leucocyte Antigen-B27 (HLA-B27) is most closely related to the genetic susceptibility of AS. In addition, there are many genes associated with AS, such as endoplasmic reticulum aminopeptidase (ERAP) and interleukin (IL) [3]. AS, as a type of spondyloarthropathy (SpA), results in 70% of AS patients having intestinal mucosal inflammation. Additionally, axial SpA is clinically linked to reactive arthritis, psoriasis, or inflammatory bowel illness in roughly 15–20% of cases [4]. Body immunity is also involved in the progression of AS. The incidence of peripheral arthritis in AS patients is significantly correlated with anti-cyclic peptide containing citrulline (anti-CCP) [5]. The abnormal expression of T cell co-stimulator CD154 is closely related to the incidence of AS [6]. Tumor necrosis factor-α (TNF-α), IL-23, and IL-17 play an important role in the inflammatory response of AS, and may serve as effective therapeutic targets [7,8]. There is no effective radical treatment strategy for AS, which is mainly divided into non-drug therapy, drug therapy and surgical therapy. Non-drug treatment mainly pursues health education regarding the patient’s psychology and lifestyle, and physical therapy. Drug treatment mainly includes nonsteroidal anti-inflammatory drugs (NSAIDs) [9], disease modifying antirheumatic drugs (DMARDs) that including conventional synthetic DMARDs (csDMARDs), biologics DMARDs (bDMARDs) and targeted synthetic DMARDs (tsDMARDs) [10], and glucocorticoids [11], etc. Additionally, NSAIDs is the first-line treatment drug for AS, which is mainly used to improve the symptoms of patients [9]. For patients with AS who do not respond to non-surgical treatment, surgical treatment can be used [12,13]. AS has a long course of disease, refractory delay, high disability rate, and the toxic side effects of long-term drug treatment and high treatment costs, which seriously affect the physical and mental health of patients and bring a huge burden to the family and society.

The gastrointestinal tract is an important metabolic organ where a large number of microorganisms gather. The gut microbiota is a community of bacteria inhabiting the human gut, which is interdependent with the human body throughout the life process. In recent years, studies have found that gut microbiota is associated with immune, metabolic and neurological properties, drug metabolism, and cancer [14]. The gut microbiota is involved in nutrient processing, immune system development, colonization resistance and the stimulation of various host activities, which is very important for maintaining human health and maintaining the dynamic balance of the body [15]. Gut microbiota is associated with many types of disease. Gut microbiota is not only associated with autism, mood disorders and other psychiatric diseases [16], but also with blood diseases such as deep vein thrombosis [14]. In addition, the gut microbiota is also associated with orthopedic diseases such as osteoarthritis and rheumatoid arthritis [17,18]. The human body’s “invisible organ”, or gut microbiota, is becoming more widely recognized as being crucial to host health [19]. It is necessary for us to link gut microbiota with human diseases and find meaningful breakthroughs in disease prevention, diagnosis and treatment from gut microbiota.

According to studies, AS and gut microbiota are tightly connected. Untreated AS patients have altered gut microbiota with diagnostic potential, and some AS-enriched species may act as molecular mimics to initiate autoimmunity [20]. The gut microbiota of AS patients is significantly different from that of healthy people, and the microbiota of AS patients is related to dietary factors and disease activity [21]. It was found that smoking and TNF-α-blockers had significant effects on the composition, relative abundance and diversity of gut microbiota in AS patients [22]. In animal experiments, it was found that changes in the gut microbiota of mice could delay the progression of AS [23,24]. Previous studies have found that a variety of gut microbiota are associated with AS, but the gut microbiotas that are more studied, relatively, with AS include Bacteroides, Streptococcus, Proteobacteria and Lachnospiraceae, etc. [20,25,26,27,28,29,30,31]. Although these four gut microbiotas are common gut microbiota associated with AS, there is no clear conclusion on their abundance level in AS. Therefore, further research on their correlation with AS is potentially valuable for the prevention, diagnosis and treatment of AS.

Mendelian Randomization (MR) research design follows the Mendelian genetic law of “parental alleles are randomly assigned to offspring”. If genotype determines phenotype, then genotype is associated with disease through phenotype. Thus, genotypes can be used as instrumental variables (IVs) to infer associations between phenotypes and diseases. The MR method uses genetic variation as an IV to build a model and deduce the causal effect. In epidemiological studies, the existence of confounding factors has greatly interfered with the causal inference of exposure and outcome. In theory, the MR method can effectively avoid the influence of confounding factors and eliminate the interference of reverse causality. At present, the MR method has been widely used to assess the causal relationship between traits and diseases and between diseases. For example, MR studies have found that there are no genetic causal relationships between vitamin-D and AS [32], but there are genetic causal relationships between inflammatory bowel disease and AS [33]. An MR study which explored the causal relationship between physical activity and AS found that there is a negative causal relationship between total physical activity and AS, while vigorous physical activity or moderate to vigorous physical level had no causal relationship with AS [34]. In addition, a previous study found a genetic causal relationship between gut microbiota and deep vein thrombosis [14]. In this study, we discussed the causal relationship between Bacteroides, Streptococcus, Proteobacteria and Lachnospiraceae and AS from the genetic level through the MR analysis, which contributes actively to the further study of AS.

## 2. Materials and Methods

### 2.1. Study Design

Based on the genome-wide association study (GWAS) summary data of gut microbiota and AS, this study screened eligible IVs for MR analysis to explore the causal relationship between gut microbiota and AS. This study strictly followed the three assumptions of MR analysis: (1) The IVs selected were related with exposure; (2) IVs were not related to any confounder factors; (3) IVs can affect outcomes only through exposure. All datasets used in this study are publicly available. Ethical permission and written informed consent had been provided in the initial studies.

### 2.2. GWAS Summary Data for Gut Microbiota

GWAS summary data of gut microbiota (Bacteroides, Streptococcus, Proteobacteria, Lachnospiraceae) were obtained from the MiBioGen consortium (www.mibiogen.org, accessed on 5 October 2022). There were 14,306 samples, all of whom were of European descent, and informed consent was provided. Bacteroides has 5,729,148 SNPs, Streptococcus has 5,643,866 SNPs, Proteobacteria has 5,728,442 SNPs, and Lachnospiraceae has 5,729,268 SNPs. Used the HRC 1.0 or 1.1 reference panel and the Michigan Imputation Server (https://imputationserv-er.sph.umich.edu/index.html) for imputation [35]. VCFs from post-imputation were converted to TriTyper format and filtered using GenotypeHarmonizer software (v.1.4.20). To procure the distributions under the null hypothesis, we used SNPSNAP to find the best 1000 SNPs that matched each upper SNP in terms of allele frequency, gene density, number of linkage disequilibrium (LD) pairs, and length from the nearest gene [36]. The Spearman correlation was used to find loci that influenced the covariate-adjusted abundant supply of bacterial taxa, excluding samples with zero abundance. As little more than a powerful approach for bitwise traits GWAS, we had to use a two-stage approach consisting of fast covariance registered by logistic regression analysis. The weighted z-score method was incorporated in Bi-naryMetaAnalyzer (v.1.0.13B available on MiBioGen Cookbook), a component of the eQTL mapping pipeline for use in vast eQTL meta-analyses [37]. More details on the data are available in published studies [38].

### 2.3. GWAS Summary Data for AS

GWAS summary data of AS were obtained from the FinnGen consortium (https://www.r7.finngen.fi/, accessed on 5 October 2022). All participants were of European ancestry, and informed consent was provided. The FinnGen research project integrates genetic data of disease endpoint from the Finnish Biobank and the Finnish Health Registry [39]. The FinnGen research project aimed to identify genotype–phenotype correlations in the Finnish population. There were 1462 AS patients and 164,682 controls, and 16,380,022 SNPs were included. All cases were defined using the M13 code in the International Classification of Diseases-Tenth Revision (ICD-10). These individuals were genotyped using Illumina (Illumina Inc, San Diego, CA, USA) and Affymetrix chip arrays (Thermo Fisher Scientific, Santa Clara, CA, USA), and 16,962,023 variants were analyzed in total. Detailed information on the participants, genotyping, imputation, and quality control can be found on the FinnGen website (https://finngen.gitbook.io/documentation/, accessed on 5 October 2022).

### 2.4. IV Selection

We performed a series of strict quality controls to select IVs to meet the three assumptions of MR analysis and ensure the robustness and reliability of MR analysis. First, we obtained SNPs related to four gut microbiotas (Bacteroides, Streptococcus, Proteobacteria, Lachnospiraceae) (*p* < 1 × 10^−5^) [40,41]. Second, the LD between SNPs was eliminated because strong LD could lead to biased results (r2 < 0.001, clumping distance = 10,000 kb) [39]. Third, we excluded SNPs associated with AS (*p* < 1 × 10^−5^). Fourth, we applied the PhenoScanner database (http://www.phenoscanner.medschl.cam.ac.uk/phenoscanner, accessed on 7 October 2022) to exclude confounder factors [42]. The main risk factors for AS were obesity, body mass index (BMI), smoking and diabetes, identified through the literature research [34,43,44,45]. Fifth, to satisfy the strong association with exposure, we selected SNPs with F statistic >10 as IVs. F statistics were calculated using the formula F = R^2^(N − K − 1)/K(1 − R^2^), and R^2^ was calculated using the formula R^2^ = (2 × EAF × (1 − EAF) × Beta^2^)/[(2 × EAF × (1 − EAF) × Beta^2^) + (2 × EAF × (1 − EAF) × N × SE^2^)] [46,47]. Sixth, for ensuring the accuracy of the results, palindromic SNPs with intermediary allele frequencies were deleted [48]. Seventh, in the case that SNPs in GWAS were not obtainable, proxy SNPs were obtained by the LDlink online platform (https://ldlink.nci.nih.gov/).

### 2.5. Statistical Analysis

Based on the IVs selected above, we performed a two-sample MR analyses of gut microbiota and AS through the TwoSampleMR and MRPRESSO packages in R (version 4.1.2). We carried out the MR analysis in five different ways: the random-effects inverse variance weighted (IVW) as the main method, and MR Egger, weighted median, simple mode, and weighted mode as supplementary methods. Random-effects IVW results are the main basis of our study. While every genetic variant fulfills the IVs assumptions, the IVW methodology employs a meta-analysis method to incorporate the Wald ratio assessments of the causal connection generated from various SNPs and produces a reliable assessment of the causal connection of the exposure on the outcome [49]. The MR Egger was not dependent on nonzero mean pleiotropy, but it reduces statistical power [50]. Weighted medians can provide strong estimates of effective IVs with at least a 50% weight [51]. The simple mode is a model-based assessment approach that offers pleiotropy robustness [52]. For mode assessment, the weighted mode is sensitive to the hard throughput collection [53].

The Cochran’s Q statistic (MR-IVW) and Rucker’s Q statistic (MR Egger) were used to detect the heterogeneity of our MR analysis, and *p* > 0.05 indicated no heterogeneity [54]. The intercept test of MR Egger was used to detect the horizontal pleiotropy, and *p* > 0.05 indicated no horizontal pleiotropy [42]. In addition, the MR pleiotropy residual sum and outlier (MR-PRESSO) can not only detect horizontal pleiotropy, but also identify outliers [42]. The “Leave one out” analysis was used to investigate whether the causal relationship between gut microbiota and AS was influenced by a single SNP [49]. The global test of MR-PRESSO analysis was used to perform the horizontal pleiotropy test, and *p* > 0.05 indicated no horizontal pleiotropy, and the distortion test of MR-PRESSO analysis was used to detect whether there were outliers in our MR analysis [55]. Importantly, any outliers in the distortion test of MR-PRESSO analysis were excluded and the causal estimates were reassessed. Furthermore, we applied the radial variants of the IVW and MR Egger model to visualize the causal estimates, and they also can detect the outliers [56]. Moreover, the MR robust adjusted profile score (MR-RAPS) method was used to assess the causal relationship and validate the robustness of MR analysis [56]. The *p* > 0.05 indicated that it conformed to the normal distribution and the evaluation results had strong robustness. Finally, the maximum likelihood, penalized weighted median, and IVW (fixed effects) were used as validation methods to further identify the potential causal association between gut microbiota and AS.

## 3. Results

### 3.1. IVs Selection

Through screening for SNPs associated with exposure and removing LD, we obtained 12 SNPs associated with Bacteroides, 17 SNPs associated with Streptococcus, 14 SNPs associated with Proteobacteria, and 19 SNPs associated with Lachnospiraceae. We further identified 12 SNPs shared by Bacteroides and AS and excluded one SNP (rs28757219) associated with AS, there were no confounding SNPs, the 11 SNPs were used as IVs (F statistic >10), and there were two palindromic SNPs (rs2366421, rs495004). We further identified 16 SNPs shared by Streptococcus and AS, there were no SNPs associated with AS or confounding, the 16 SNPs were used as IVs (F statistic > 10), and there were two palindromic SNPs (rs395407, rs6563952). We further identified 14 SNPs shared by Proteobacteria and AS, there were no SNPs associated with AS or confounding, the 14 SNPs were used as IVs (F statistic > 10), and there were two palindromic SNPs (rs312757, rs74757828). We further identified 17 SNPs shared by Lachnospiraceae and AS, there were no SNPs associated with AS or confounding, the 17 SNPs were used as IVs (F statistic > 10), and there was one palindromic SNP (rs11755180) (Supplementary File).

### 3.2. MR Analysis

**Figure 1:**
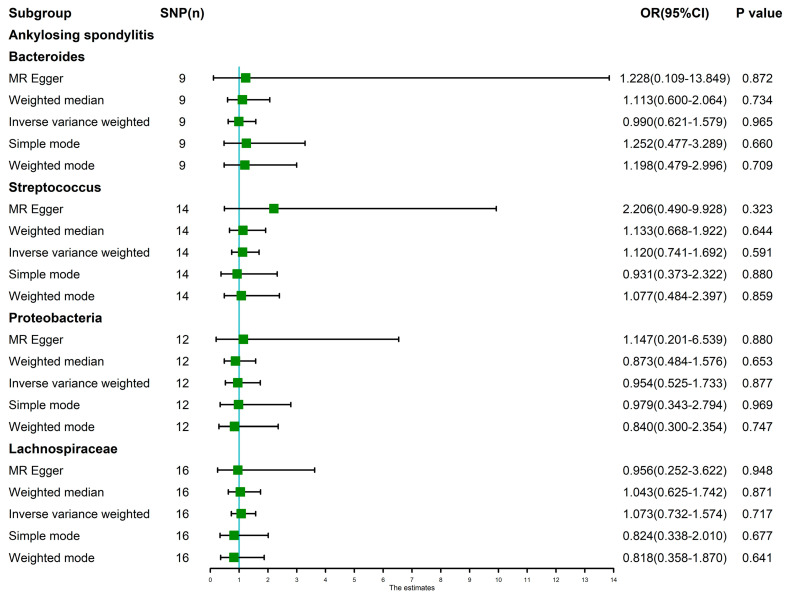
MR analysis between the gut microbiota (Bacteroides, Streptococcus, Proteobacteria and Lachnospiraceae) and ankylosing spondylitis. Five methods: random-effects IVW, MR Egger, weighted median, simple mode, and weighted mode.

**Figure 2:**
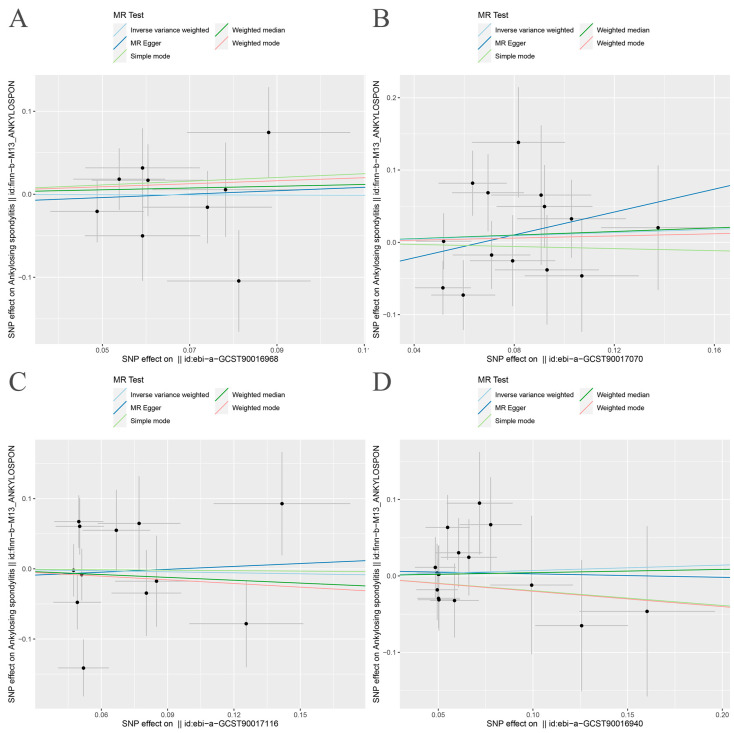
Scatter plot of MR analysis. (**A**) Bacteroides and ankylosing spondylitis; (**B**) Streptococcus and ankylosing spondylitis; (**C**) Proteobacteria and ankylosing spondylitis; (**D**) Lachnospiraceae and ankylosing spondylitis.

The random-effects IVW results showed that Bacteroides (*p* = 0.965, OR 95% confidence interval [CI] = 0.990 [0.621–1.579]), Streptococcus (*p* = 0.591, OR 95% CI = 1.120 [0.741–1.692]), Proteobacteria (*p* = 0.877, OR 95% CI = 0.954 [0.525–1.733]) and Lachnospiraceae (*p* = 0.717, OR 95% CI = 1.073 [0.732–1.574]) had no genetic causal relationship with AS. The analysis results of MR Egger, weighted median, simple mode and weighted mode were consistent with random-effects IVW (Figure 1 and Figure 2).

**Figure 3:**
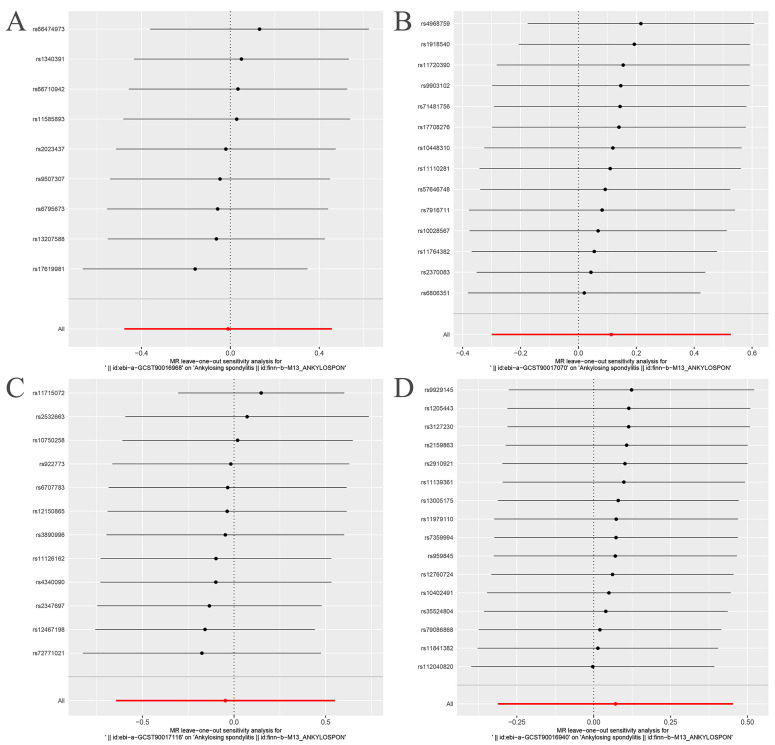
“Leave one out” analysis. The red lines are the analysis results of random effects IVW. (**A**) Bacteroides and ankylosing spondylitis; (**B**) Streptococcus and ankylosing spondylitis; (**C**) Proteobacteria and ankylosing spondylitis; (**D**) Lachnospiraceae and ankylosing spondylitis.

**Figure 4:**
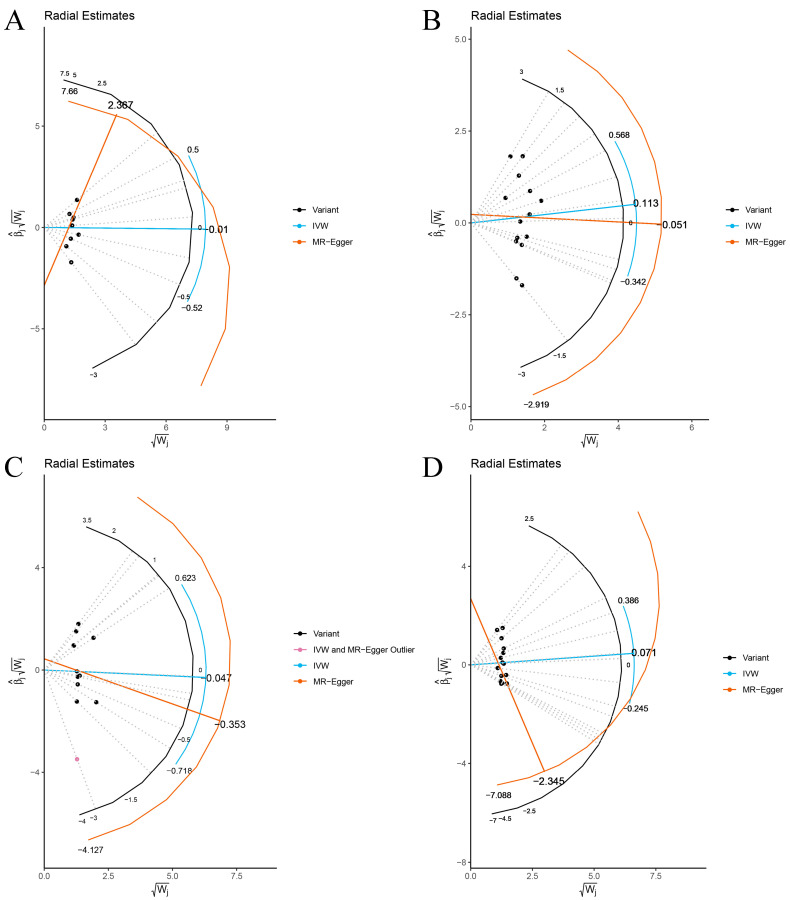
The MR estimate visualizes the radial plot of a single outlier SNPs, and the curve shows the ratio estimate of each SNP. Black dots show valid SNPs. (**A**) MR radial plots of Bacteroides and ankylosing spondylitis; (**B**) MR radial plots of Streptococcus and ankylosing spondylitis; (**C**) MR radial plots of Proteobacteria and ankylosing spondylitis; (**D**) MR radial plots of Lachnospiraceae and ankylosing spondylitis.

**Figure 5:**
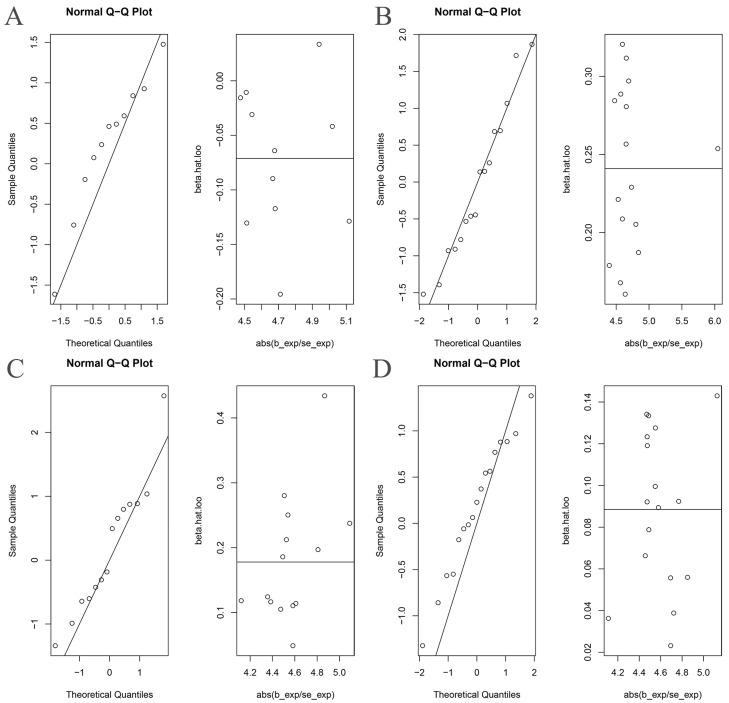
The normal distribution plots of MR analysis for gut microbiota and ankylosing spondylitis. (**A**) Bacteroides and ankylosing spondylitis; (**B**) Streptococcus and ankylosing spondylitis; (**C**) Proteobacteria and ankylosing spondylitis; (**D**) Lachnospiraceae and ankylosing spondylitis.

The Cochran’s Q statistic (MR-IVW) and Rucker’s Q statistic (MR Egger) showed that the MR analyses of Bacteroides, Streptococcus and Lachnospiraceae and AS had no heterogeneity (*p* > 0.05), and the MR analysis of Proteobacteria and AS had heterogeneity (*p* < 0.05) (Table 1). The intercept test of MR Egger analysis showed that the MR analyses of Bacteroides, Streptococcus, Proteobacteria and Lachnospiraceae and AS had no horizontal pleiotropy (*p* > 0.05) (Table 1). The “Leave one out” analysis indicated that our MR analyses were not driven by a single SNP (Figure 3). The global test of MR-PRESSO showed that the MR analyses of Bacteroides, Streptococcus and Lachnospiraceae and AS had no horizontal pleiotropy (*p* > 0.05), and the MR analysis of Proteobacteria and AS had horizontal pleiotropy (*p* < 0.05) (Table 1). The distortion test of MR-PRESSO analysis showed that the MR analyses of Bacteroides, Streptococcus and Lachnospiraceae and AS had no outliers, and the MR analysis of Proteobacteria and AS had one outlier (rs11715072) (Table 1). In addition, the IVW and MR Egger radial MR method delineation showed that there were no outliers in the MR analyses between Bacteroides, Streptococcus, Lachnospiraceae and AS, and there was one outlier in the MR analysis between Proteobacteria and AS (Figure 4). The MR-RAPS analysis showed that Bacteroides (*p* = 0.765, OR = 0.931), Streptococcus (*p* = 0.232, OR = 1.273), Proteobacteria (*p* = 0.502, OR = 1.195) and Lachnospiraceae (*p* = 0.668, OR = 1.093) had no genetic causal relationship with AS (Table 1). In addition, the MR-RAPS showed that the MR analyses between Bacteroides, Streptococcus, Proteobacteria, Lachnospiraceae and AS were normally distributed (*p* > 0.05) (Table 1, Figure 5).

**Figure 6:**
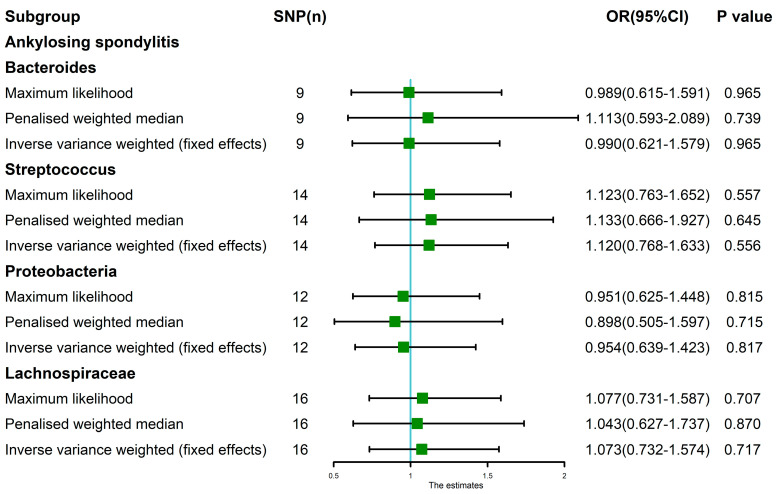
The MR analysis between gut microbiota (Bacteroides, Streptococcus, Proteobacteria and Lachnospiraceae) and ankylosing spondylitis. Three methods: maximum likelihood, penalized weighted median and IVW (fixed effects).

The maximum likelihood, penalized weighted median and IVW (fixed effects) results showed that Bacteroides, Streptococcus, Proteobacteria and Lachnospiraceae had no genetic causal relationship with AS (*p* > 0.05) (Figure 6).

### 3.3. MR Analysis after Removing Outliers

As there were heterogeneity and horizontal pleiotropy in the MR analysis of Proteobacteria and AS, and one outlier (rs11715072) was detected by MR-PRESSO, after eliminating the outliers we ran a second round of MR analysis.

**Figure 7:**
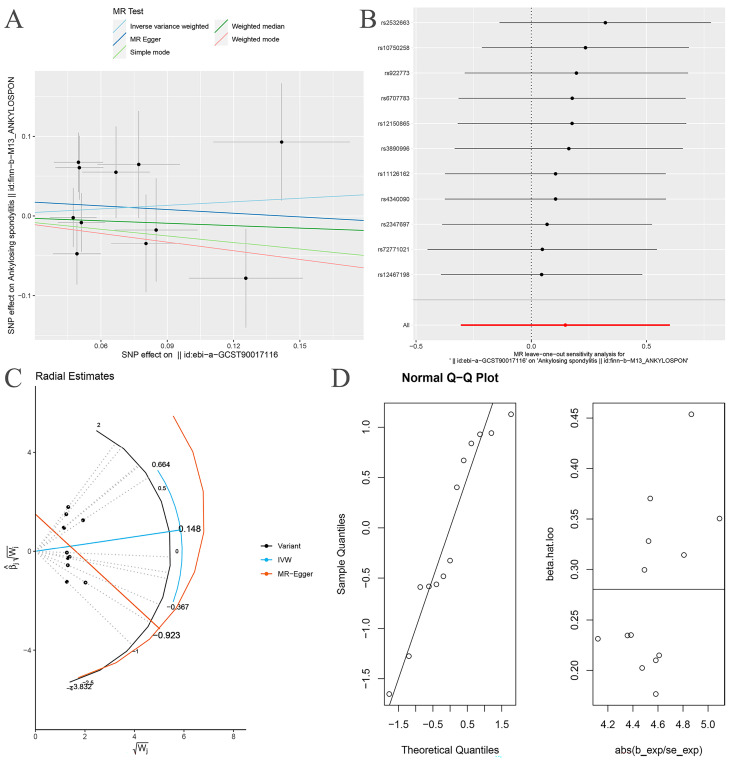
MR analysis of the Proteobacteria and ankylosing spondylitis after exclusion of one outlier SNP (rs11715072). (**A**) Scatter plot; (**B**) “Leave one out” analysis, the red lines are the analysis results of random effects IVW; (**C**) MR radial plots; (**D**) the normal distribution plots.

**Figure 8:**
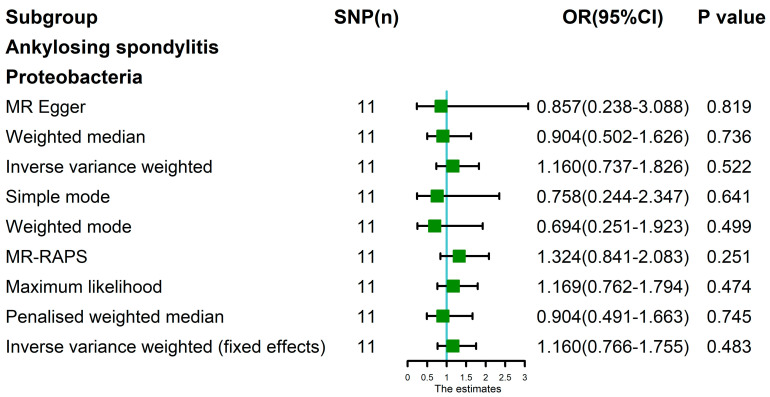
MR analysis of the Proteobacteria and ankylosing spondylitis after exclusion of one outlier SNP (rs11715072). Nine methods: MR Egger, weighted median, random-effects IVW, simple mode, weighted mode, MR-RAPS, maximum likelihood, penalized weighted median, and IVW (fixed effects).

After removing rs11715072, the random-effects IVW results showed that there was no genetic causal relationship between Proteobacteria and AS (*p* = 0.522, OR 95% CI = 1.160 [0.737–1.826]) (Figure 7A and Figure 8). The Cochran’s Q statistic (MR-IVW) and the Rucker’s Q statistic (MR Egger) showed that the MR analysis of Proteobacteria and AS had no heterogeneity (*p* > 0.05). The intercept test of MR Egger showed that the MR analysis of Proteobacteria and AS had no horizontal pleiotropy (*p* > 0.05) (Table 1). The “Leave one out” analysis indicated that the MR analysis results of Proteobacteria and AS were not driven by a single SNP (Figure 7B). The global test of MR-PRESSO analysis showed that the MR analysis of Proteobacteria and AS had no horizontal pleiotropy (*p* > 0.05), and the distortion test of MR-PRESSO analysis showed that the MR analysis of Proteobacteria and AS had no outliers (Table 1). In addition, the IVW and MR Egger radial MR method delineation showed that there were no outliers in the MR analysis between Proteobacteria and AS (Figure 7C). The MR-RAPS analysis showed that the MR analyses between Proteobacteria and AS were normally distributed (*p* > 0.05) (Table 1, Figure 7D).

The MR analysis results of MR Egger, weighted median, wimple mode, Weighted mode, MR-RAPS, maximum likelihood, penalized weighted median, and IVW (fixed effects) were consistent with random-effects IVW (Table 1, Figure 8).

## 4. Discussion

In this study, we used the MR method to analyze the genetic causal correlation between four common gut microbiotas associated with AS and AS. The MR method conducted causal analysis of the four gut microbiotas and AS from the genetic level, effectively avoiding the trouble caused by traditional observational research. Our results showed that Bacteroides, Streptococcus, Proteobacteria and Lachnospiraceae were not causally correlated with AS at the genetic level, but it could not be ruled out that gut microbiota and AS were related at other levels except genetics. MR analysis explores the relationship between the two from the genetic level, and MR analysis can directly reveal the genetic causal relationship between traits and diseases or diseases. Therefore, what our study reveals is the genetic causal relationship between the gut microbiotas in the human body and AS patients, rather than the host genome. If the results of this study show that a certain gut microbiota has a positive or negative genetic causal relationship with AS, it can be indicated that the imbalance of this gut microbiota may be related to the occurrence and development of AS. However, there was no causal relationship between the gut microbiotas (Bacteroides, Streptococcus, Proteobacteria and Lachnospiraceae) studied in this study and AS at the genetic level, which makes a positive contribution to the genetic research of AS.

Bacteroides are one of the dominant microbiotas in the intestinal tract of human and animal, accounting for 1/4 of the total gut microbiotas. Bacteroides are a normal colonizing bacteria in the intestinal tract of mammals, but when a part of the body is damaged or has pathological changes in advance, Bacteroides can be translocated and become opportunistic pathogens. It was found that Bacteroides were the common pathogenic bacteria of soft tissue infection, abdominal abscess and bacteremia [57]. It was found that the abundance of Bacteroides in AS patients was significantly decreased [25]. The decrease of the abundance of Bacteroides may lead to the decrease of lipopolysaccharide and flagellin levels and cause the deficiency of anti-microbial peptide (Reg IIγ) secretion, which causes an imbalance of gut microbiotas and promotes the occurrence of AS [25]. There are also studies showing that Bacteroides are specifically enriched in the intestinal tract of AS patients [20]. Bacteroides fragilis is reported to be increased in febrile related arthritis in children [58]. Although Bacteroides falciparum poison has been documented to have pro-inflammatory and oncogenic effects, its polysaccharide A is linked to anti-inflammatory properties [59,60]. We speculate that the role of Bacteroides in the pathogenesis of AS patients may be related to the proinflammatory effect of Bacteroides fragilis toxin. This study found that there was no genetic causal relationship between Bacteroides and AS. At present, there is no clear conclusion on the abundance of Bacteroides in AS. The results of this study at least show that such uncertainty has nothing to do with genetic factors, and we cannot exclude the correlation between Bacteroides and AS in other aspects besides genetics.

Streptococcus is a diversified genus with 104 taxonomic groups, and its conversations with the host genome range from symbionts to pathogenic. Many pathogens cause serious, life-threatening infections that have a significant mortality and morbidity burden [61]. Streptococcus can cause a variety of diseases in clinic, such as lobar pneumonia, otitis media, bronchitis, meningitis and septicemia [62]. The studies found an increased abundance of Streptococcus in patients with AS [26,29,31]. Streptococcus salivarius (S.alivarius) ATCC 25975 was found to have pro-inflammatory properties [63]. In addition, Streptococcus genus infection is associated with reactive arthritis [26]. However, other studies have found that Streptococcus salivarius can inhibit the nuclear factor kappa-B (NF-κB) pathway and down-regulate the secretion of IL-8 in human intestinal epithelial cells [64]. Combined with the results of our study, we considered that there was no causal relationship between Streptococcus and AS at the genetic level. Previous studies have shown that Streptococcus has an increased abundance in AS patients, and Streptococcus may promote the disease progression in AS patients to a certain extent. This may be related to the pro-inflammatory property of Streptococcus, and the correlation between Streptococcus and AS may be influenced by other factors besides genetics.

Proteobacteria are very common microbiotas in the human intestine. Most of them are facultative anaerobe and Gram-negative bacteria. They can produce lipopolysaccharide and facultative flagellin to promote inflammation and have a certain pathogenicity. When the anaerobic environment of the colon is damaged, facultative anaerobes (mainly Proteobacteria) will rapidly multiply and compete with the host for nutrients using short chain fatty acids (SCFAs). At the same time, the abundance of obligate anaerobes will be reduced due to the influence of oxygen. In addition, SCFAs can promote the differentiation of initial T cells into regulatory T cells (Treg) by inhibiting the activity of histone deacetylase, thus affecting intestinal epigenetic enzymes [65]. Studies have shown that SCFAs can promote the differentiation of Treg while inhibiting the differentiation of T helper cell 17 (Th17) [66]. Additionally, more importantly, Foxp3+ Treg plays an important role in inhibiting the intestinal inflammation of AS [65]. It can be seen that the imbalance of Proteobacteria plays a certain role in the occurrence and development of AS. However, according to our study, there is no causal relationship between Proteobacteria and AS at the genetic level. Compared with the results of other studies, the role of Proteobacteria in AS is mainly related to metabolism and other relevant factors.

The primary group of gut microbes, Lachnospiraceae, populates the gut lumen from birth and multiplies throughout the host’s lifetime in the sense of species richness and relative amounts. Different species of the Lachnospiraceae are linked to a variety of intra- and extraintestinal illnesses, despite the fact that they are among the main suppliers of relatively brief fatty acids [67]. It was found that the gut microbiota structure of AS patients was significantly changed compared with the normal population, and the abundance of Lachnospiraceae in the terminal ileum of AS patients was higher than that of healthy controls [67]. Some studies also found that the abundance of Lachnospiraceae decreased in AS patients [29]. Although this differs from our findings, we think it is reasonable. Lachnospiraceae has been shown in studies to be able to control the host’s immunological state by suppressing the synthesis of TNF-α and raising the production of IL-10 [67,68]. Since TNF-α can effectively inhibit the occurrence of AS [7,8], we speculated that Lachnospiraceae may affect the occurrence and development of AS by regulating immune metabolites. These studies provide a reference for the differences between our research results and previous studies. We have reason to consider that Lachnospiraceae, which has no causal relationship with AS at the genetic level, plays a certain role in the occurrence and development of AS, because Lachnospiraceae affects AS by regulating the immune state of the host.

Of course, this study also had some limitations. First, our study only analyzed populations from Europe, and we need to use our conclusions with caution when we extend them to other populations. Secondly, gender was not differentiated in our study. For our results, it is necessary to consider whether there would be differences when applied to male or female populations alone. In addition, we only analyzed the gut microbiota with more studies on AS, and the number of included gut microbiota was small, which was not enough to represent the entire gut microbiota. Finally, our study was only conducted at the genetic level, and did not study whether the special structure and metabolites of the bacteria had a huge impact on the occurrence and development of AS. At the same time, the gut microbiota is complex and diverse, and our study did not consider whether AS would be affected by the complex interrelationships among various types of microbiotas in the case of gut microbiota imbalance.

## 5. Conclusions

Our research results showed that there were no causal relationships between Bacteroides, Streptococcus, Proteobacteria and Lachnospiraceae and AS at the genetic level. However, that does not rule out the possibility that they are related on a level other than genetic. Deeper and more extensive research is needed to investigate these possible links.

## Figures and Tables

**Table 1:** Sensitivity analysis of the MR analysis results of exposures and outcomes.

Exposure	Outcome	Heterogeneity Test		Pleiotropy Test	MR-PRESSO		MR-RAPS		
		Cochran’s Q Test (*p* Value)	Rucker’s Q Test (*p* Value)	Egger Intercept (*p* Value)	Distortion Test	Global Test	MR Analysis		Normal Distribution
		IVW	MR-Egger	MR-Egger	Outliers	*p* Value	OR	*p* Value	*p* Value
Bacteroides	AS	0.547	0.443	0.863	NA	0.667	0.931	0.765	0.569
Streptococcus	AS	0.272	0.266	0.376	NA	0.330	1.273	0.232	0.548
Proteobacteria	AS	0.011	0.007	0.828	1	0.014	1.195	0.502	0.416
Proteobacteria *	AS	0.286	0.233	0.631	NA	0.298	1.324	0.251	0.187
Lachnospiraceae	AS	0.897	0.857	0.861	NA	0.908	1.093	0.668	0.905

AS: ankylosing spondylitis; Proteobacteria *: MR analysis of the Proteobacteria and ankylosing spondylitis after exclusion of one outlier SNP (rs11715072).

## Data Availability

Publicly available datasets were analyzed in this study. These data can be found here: MiBioGen consortium (www.mibiogen.org, accessed on 5 October 2022) and FinnGen consortium (https://www.r7.finngen.fi/, accessed on 5 October 2022).

## Supplementary Materials

The following supporting information can be downloaded at: https://www.mdpi.com/article/10.3390/nu15041057/s1, Supplementary File: Instrumental variables for Mendelian randomization analysis of ankylosing spondylitis and gut microbiota.

## References

[1] Zhu W., He X., Cheng K., Zhang L., Chen D., Wang X., Qiu G., Cao X., Weng X. (2019). Ankylosing spondylitis: Etiology, pathogenesis, and treatments. Bone Res..

[2] Exarchou S., Lindström U., Askling J., Eriksson J.K., Forsblad-d’Elia H., Neovius M., Turesson C., Kristensen L.E., Jacobsson L.T. (2015). The prevalence of clinically diagnosed ankylosing spondylitis and its clinical manifestations: A nationwide register study. Arthritis Res. Ther..

[3] Pedersen S.J., Maksymowych W.P. (2019). The Pathogenesis of Ankylosing Spondylitis: An Update. Curr. Rheumatol. Rep..

[4] Sieper J., Poddubnyy D. (2017). Axial spondyloarthritis. Lancet.

[5] Kim J.O., Lee J.S., Choi J.Y., Lee K.H., Kim Y.B., Yoo D.H., Kim T.H. (2013). The relationship between peripheral arthritis and anti-cyclic citrullinated peptide antibodies in ankylosing spondylitis. Joint Bone Spine.

[6] Lin Q., Lin Z., Gu J., Huang F., Li T., Wei Q., Liao Z., Cao S., Jiang Y., Huang J. (2010). Abnormal high-expression of CD154 on T lymphocytes of ankylosing spondylitis patients is down-regulated by etanercept treatment. Rheumatol. Int..

[7] Sieper J., Poddubnyy D., Miossec P. (2019). The IL-23-IL-17 pathway as a therapeutic target in axial spondyloarthritis. Nat. Rev. Rheumatol..

[8] Furue K., Ito T., Furue M. (2018). Differential efficacy of biologic treatments targeting the TNF-α/IL-23/IL-17 axis in psoriasis and psoriatic arthritis. Cytokine.

[9] Song I.H., Poddubnyy D.A., Rudwaleit M., Sieper J. (2008). Benefits and risks of ankylosing spondylitis treatment with nonsteroidal antiinflammatory drugs. Arthritis Rheum..

[10] Chiu Y.M., Chen D.Y. (2020). Infection risk in patients undergoing treatment for inflammatory arthritis: Non-biologics versus biologics. Expert Rev. Clin. Immunol..

[11] Siu S., Haraoui B., Bissonnette R., Bessette L., Roubille C., Richer V., Starnino T., McCourt C., McFarlane A., Fleming P. (2015). Meta-analysis of tumor necrosis factor inhibitors and glucocorticoids on bone density in rheumatoid arthritis and ankylosing spondylitis trials. Arthritis Care Res..

[12] Lin D., Charalambous A., Hanna S.A. (2019). Bilateral total hip arthroplasty in ankylosing spondylitis: A systematic review. EFORT Open Rev..

[13] Wang T., Zheng G., Wang Y., Zhang X., Hu F., Wang Y. (2019). Comparison of 2 Surgeries in Correction of Severe Kyphotic Deformity Caused by Ankylosing Spondylitis: Vertebral Column Decancellation and Pedicle Subtraction Osteotomy. World Neurosurg..

[14] Yang M., Luo P., Zhang F., Xu K., Feng R., Xu P. (2022). Large-scale correlation analysis of deep venous thrombosis and gut microbiota. Front. Cardiovasc. Med..

[15] Gosalbes M.J., Durban A., Pignatelli M., Abellan J.J., Jimenez-Hernandez N., Perez-Cobas A.E., Latorre A., Moya A. (2011). Metatranscriptomic approach to analyze the functional human gut microbiota. PLoS ONE.

[16] Mangiola F., Ianiro G., Franceschi F., Fagiuoli S., Gasbarrini G., Gasbarrini A. (2016). Gut microbiota in autism and mood disorders. World J. Gastroenterol..

[17] Liu Y., Ding W., Wang H.L., Dai L.L., Zong W.H., Wang Y.Z., Bi J., Han W., Dong G.J. (2019). Gut microbiota and obesity-associated osteoarthritis. Osteoarthr. Cartil..

[18] Mei L., Yang Z., Zhang X., Liu Z., Wang M., Wu X., Chen X., Huang Q., Huang R. (2021). Sustained Drug Treatment Alters the Gut Microbiota in Rheumatoid Arthritis. Front. Immunol..

[19] Hill C.J., Lynch D.B., Murphy K., Ulaszewska M., Jeffery I.B., O’Shea C.A., Watkins C., Dempsey E., Mattivi F., Tuohy K. (2017). Evolution of gut microbiota composition from birth to 24 weeks in the INFANTMET Cohort. Microbiome.

[20] Zhou C., Zhao H., Xiao X.Y., Chen B.D., Guo R.J., Wang Q., Chen H., Zhao L.D., Zhang C.C., Jiao Y.H. (2020). Metagenomic profiling of the pro-inflammatory gut microbiota in ankylosing spondylitis. J. Autoimmun..

[21] Zhang L., Han R., Zhang X., Fang G., Chen J., Li J., Xu S., Qian L., Chen W., Pan F. (2019). Fecal microbiota in patients with ankylosing spondylitis: Correlation with dietary factors and disease activity. Clin. Chim. Acta.

[22] Zhang F., Ma C., Zhang B., Bi L. (2020). Dynamic changes in gut microbiota under the influence of smoking and TNF-alpha-blocker in patients with ankylosing spondylitis. Clin. Rheumatol..

[23] Liu B., Yang L., Cui Z., Zheng J., Huang J., Zhao Q., Su Z., Wang M., Zhang W., Liu J. (2019). Anti-TNF-α therapy alters the gut microbiota in proteoglycan-induced ankylosing spondylitis in mice. Microbiologyopen.

[24] Yang L., Liu B., Zheng J., Huang J., Zhao Q., Liu J., Su Z., Wang M., Cui Z., Wang T. (2019). Rifaximin Alters Intestinal Microbiota and Prevents Progression of Ankylosing Spondylitis in Mice. Front. Cell. Infect. Microbiol..

[25] Wen C., Zheng Z., Shao T., Liu L., Xie Z., Le Chatelier E., He Z., Zhong W., Fan Y., Zhang L. (2017). Quantitative metagenomics reveals unique gut microbiome biomarkers in ankylosing spondylitis. Genome Biol..

[26] Sternes P.R., Brett L., Phipps J., Ciccia F., Kenna T., de Guzman E., Zimmermann K., Morrison M., Holtmann G., Klingberg E. (2022). Distinctive gut microbiomes of ankylosing spondylitis and inflammatory bowel disease patients suggest differing roles in pathogenesis and correlate with disease activity. Arthritis Res. Ther..

[27] Liu B., Ding Z., Xiong J., Heng X., Wang H., Chu W. (2022). Gut Microbiota and Inflammatory Cytokine Changes in Patients with Ankylosing Spondylitis. Biomed. Res. Int..

[28] Li M., Dai B., Tang Y., Lei L., Li N., Liu C., Ge T., Zhang L., Xu Y., Hu Y. (2019). Altered Bacterial-Fungal Interkingdom Networks in the Guts of Ankylosing Spondylitis Patients. mSystems.

[29] Klingberg E., Magnusson M.K., Strid H., Deminger A., Stahl A., Sundin J., Simren M., Carlsten H., Ohman L., Forsblad-d’Elia H. (2019). A distinct gut microbiota composition in patients with ankylosing spondylitis is associated with increased levels of fecal calprotectin. Arthritis Res. Ther..

[30] Costello M.E., Ciccia F., Willner D., Warrington N., Robinson P.C., Gardiner B., Marshall M., Kenna T.J., Triolo G., Brown M.A. (2015). Brief Report: Intestinal Dysbiosis in Ankylosing Spondylitis. Arthritis Rheumatol..

[31] Chen Z., Qi J., Wei Q., Zheng X., Wu X., Li X., Liao Z., Lin Z., Gu J. (2019). Variations in gut microbial profiles in ankylosing spondylitis: Disease phenotype-related dysbiosis. Ann. Transl. Med..

[32] Jiang J., Shao M., Wu X. (2022). Vitamin D and risk of ankylosing spondylitis: A two-sample mendelian randomization study. Hum. Immunol..

[33] Cui Z., Hou G., Meng X., Feng H., He B., Tian Y. (2020). Bidirectional Causal Associations Between Inflammatory Bowel Disease and Ankylosing Spondylitis: A Two-Sample Mendelian Randomization Analysis. Front. Genet..

[34] Hu S., Xing H., Wang X., Zhang N., Xu Q. (2022). Causal Relationships Between Total Physical Activity and Ankylosing Spondylitis: A Mendelian Randomization Study. Front. Immunol..

[35] McCarthy S., Das S., Kretzschmar W., Delaneau O., Wood A.R., Teumer A., Kang H.M., Fuchsberger C., Danecek P., Sharp K. (2016). A reference panel of 64,976 haplotypes for genotype imputation. Nat. Genet..

[36] Pers T.H., Timshel P., Hirschhorn J.N. (2015). SNPsnap: A Web-based tool for identification and annotation of matched SNPs. Bioinformatics.

[37] Westra H.J., Peters M.J., Esko T., Yaghootkar H., Schurmann C., Kettunen J., Christiansen M.W., Fairfax B.P., Schramm K., Powell J.E. (2013). Systematic identification of trans eQTLs as putative drivers of known disease associations. Nat. Genet..

[38] Kurilshikov A., Medina-Gomez C., Bacigalupe R., Radjabzadeh D., Wang J., Demirkan A., Le Roy C.I., Raygoza Garay J.A., Finnicum C.T., Liu X. (2021). Large-scale association analyses identify host factors influencing human gut microbiome composition. Nat. Genet..

[39] Chen Y., Shen J., Wu Y., Ni M., Deng Y., Sun X., Wang X., Zhang T., Pan F., Tang Z. (2022). Tea consumption and risk of lower respiratory tract infections: A two-sample mendelian randomization study. Eur. J. Nutr..

[40] Xu Q., Ni J.J., Han B.X., Yan S.S., Wei X.T., Feng G.J., Zhang H., Zhang L., Li B., Pei Y.F. (2021). Causal Relationship Between Gut Microbiota and Autoimmune Diseases: A Two-Sample Mendelian Randomization Study. Front. Immunol..

[41] Ni J.J., Xu Q., Yan S.S., Han B.X., Zhang H., Wei X.T., Feng G.J., Zhao M., Pei Y.F., Zhang L. (2021). Gut Microbiota and Psychiatric Disorders: A Two-Sample Mendelian Randomization Study. Front. Microbiol..

[42] Shu M.J., Li J.R., Zhu Y.C., Shen H. (2022). Migraine and Ischemic Stroke: A Mendelian Randomization Study. Neurol. Ther..

[43] Dulger S., Aykurt Karlibel I., Kasapoglu Aksoy M., Altan L., Sengoren Dikis O., Yildiz T. (2019). How Does Smoking Cessation Affect Disease Activity, Function Loss, and Quality of Life in Smokers With Ankylosing Spondylitis?. J. Clin. Rheumatol..

[44] Liao K.F., Kuo Y.H., Lai S.W. (2021). Diabetes mellitus in ankylosing spondylitis. Ann. Rheumatic Dis..

[45] Ortolan A., Lorenzin M., Felicetti M., Ramonda R. (2021). Do Obesity and Overweight Influence Disease Activity Measures in Axial Spondyloarthritis? A Systematic Review and Meta-Analysis. Arthritis Care Res..

[46] Chen H., Mi S., Zhu J., Jin W., Li Y., Wang T., Li Y., Fan C. (2021). No Causal Association Between Adiponectin and the Risk of Rheumatoid Arthritis: A Mendelian Randomization Study. Front. Genet..

[47] Dan Y.L., Wang P., Cheng Z., Wu Q., Wang X.R., Wang D.G., Pan H.F. (2021). Circulating adiponectin levels and systemic lupus erythematosus: A two-sample Mendelian randomization study. Rheumatology.

[48] Cao Z., Wu Y., Li Q., Li Y., Wu J. (2022). A causal relationship between childhood obesity and risk of osteoarthritis: Results from a two-sample Mendelian randomization analysis. Ann. Med..

[49] Lee Y.H. (2019). Causal association between smoking behavior and the decreased risk of osteoarthritis: A Mendelian randomization. Z. Rheumatol..

[50] Zheng C., He M.H., Huang J.R., He Y. (2021). Causal Relationships Between Social Isolation and Osteoarthritis: A Mendelian Randomization Study in European Population. Int. J. Gen. Med..

[51] Bowden J., Davey Smith G., Haycock P.C., Burgess S. (2016). Consistent Estimation in Mendelian Randomization with Some Invalid Instruments Using a Weighted Median Estimator. Genet. Epidemiol..

[52] Li C., Niu M., Guo Z., Liu P., Zheng Y., Liu D., Yang S., Wang W., Li Y., Hou H. (2022). A Mild Causal Relationship Between Tea Consumption and Obesity in General Population: A Two-Sample Mendelian Randomization Study. Front. Genet..

[53] Hartwig F.P., Davey Smith G., Bowden J. (2017). Robust inference in summary data Mendelian randomization via the zero modal pleiotropy assumption. Int. J. Epidemiol..

[54] Hemani G., Zheng J., Elsworth B., Wade K.H., Haberland V., Baird D., Laurin C., Burgess S., Bowden J., Langdon R. (2018). The MR-Base platform supports systematic causal inference across the human phenome. eLife.

[55] Meng H., Jiang L., Song Z., Wang F. (2022). Causal Associations of Circulating Lipids with Osteoarthritis: A Bidirectional Mendelian Randomization Study. Nutrients.

[56] Zhang J. (2022). Mendelian Randomization Study Implies Causal Linkage Between Telomere Length and Juvenile Idiopathic Arthritis in a European Population. J. Inflamm. Res..

[57] Brook I., Frazier E.H. (2000). Aerobic and anaerobic microbiology in intra-abdominal infections associated with diverticulitis. J. Med. Microbiol..

[58] Stoll M.L., Weiss P.F., Weiss J.E., Nigrovic P.A., Edelheit B.S., Bridges S.L., Danila M.I., Spencer C.H., Punaro M.G., Schikler K. (2018). Age and fecal microbial strain-specific differences in patients with spondyloarthritis. Arthritis Res. Ther..

[59] Purcell R.V., Pearson J., Aitchison A., Dixon L., Frizelle F.A., Keenan J.I. (2017). Colonization with enterotoxigenic Bacteroides fragilis is associated with early-stage colorectal neoplasia. PLoS ONE.

[60] Erturk-Hasdemir D., Kasper D.L. (2018). Finding a needle in a haystack: Bacteroides fragilis polysaccharide A as the archetypical symbiosis factor. Ann. N. Y. Acad. Sci..

[61] Andam C.P., Hanage W.P. (2015). Mechanisms of genome evolution of Streptococcus. Infect. Genet. Evol..

[62] Briles D.E., Paton J.C., Mukerji R., Swiatlo E., Crain M.J. (2019). Pneumococcal Vaccines. Microbiol. Spectr..

[63] Mostefaoui Y., Bart C., Frenette M., Rouabhia M. (2004). Candida albicans and Streptococcus salivarius modulate IL-6, IL-8, and TNF-alpha expression and secretion by engineered human oral mucosa cells. Cell Microbiol..

[64] Kaci G., Lakhdari O., Dore J., Ehrlich S.D., Renault P., Blottiere H.M., Delorme C. (2011). Inhibition of the NF-kappaB pathway in human intestinal epithelial cells by commensal Streptococcus salivarius. Appl. Environ. Microbiol..

[65] Chen B., Sun L., Zhang X. (2017). Integration of microbiome and epigenome to decipher the pathogenesis of autoimmune diseases. J. Autoimmun..

[66] Haghikia A., Jorg S., Duscha A., Berg J., Manzel A., Waschbisch A., Hammer A., Lee D.H., May C., Wilck N. (2015). Dietary Fatty Acids Directly Impact Central Nervous System Autoimmunity via the Small Intestine. Immunity.

[67] Vacca M., Celano G., Calabrese F.M., Portincasa P., Gobbetti M., De Angelis M. (2020). The Controversial Role of Human Gut Lachnospiraceae. Microorganisms.

[68] Wang L., Wang Y., Zhang P., Song C., Pan F., Li G., Peng L., Yang Y., Wei Z., Huang F. (2022). Gut microbiota changes in patients with spondyloarthritis: A systematic review. Semin. Arthritis Rheum..

